# ‘I Waited for It until Forever’: Community Barriers to Accessing Intellectual Disability Services for Children and Their Families in Cape Town, South Africa

**DOI:** 10.3390/ijerph17228504

**Published:** 2020-11-17

**Authors:** Siyabulela Mkabile, Leslie Swartz

**Affiliations:** 1 Department of Psychology, Stellenbosch University, Stellenbosch 7602, South Africa; lswartz@sun.ac.za; 2 Department of Psychiatry and Mental Health, University of Cape Town, Cape Town 7735, South Africa

**Keywords:** intellectual disability, children, South Africa, access to specialized intellectual disability services, poverty, inequality

## Abstract

Background: Intellectual disability is more common in low- and middle-income countries than in high-income countries. Stigma and discrimination have contributed to barriers to people with intellectual disability accessing healthcare. As part of a larger study on caregiving of children with intellectual disability in urban Cape Town, South Africa, we interviewed a sub-group of families who had never used the intellectual disability services available to them, or who had stopped using them. Methods: We employed a qualitative research design and conducted semi-structured interviews to explore the views and perspectives of parents and caregivers of children with intellectual disability who are not using specialised hospital services. We developed an interview guide to help explore caregivers’ and parents’ views. Results: Results revealed that caregivers and parents of children with intellectual disability did not use the intellectual disability service due to financial difficulties, fragile care networks and opportunity costs, community stigma and lack of safety, lack of faith in services and powerlessness at effecting changes and self-stigmatisation. Conclusion: Current findings highlight a need for increased intervention at community level and collaboration with community-based projects to facilitate access to services, and engagement with broader issues of social exclusion.

## 1. Introduction

While significant improvements have been observed in the health system in South Africa since the beginning of democracy, it appears the majority of vulnerable South Africans from low socio-economic backgrounds are still struggling as a result of structural disadvantage. For people with intellectual disabilities (ID), access to specialised intellectual disability services for their basic healthcare needs is still a major challenge because of structural barriers further complicated by disability. These structural barriers include difficulty accessing services for cost and distance reasons, unsafe living conditions and transport routes, poorer access to education, work and nutrition and living in conditions of environmental degradation. According to the World Health Organization, intellectual disability (ID) is defined as follows: “Intellectual disability means a significantly reduced ability to understand new or complex information and to learn and apply new skills (impaired intelligence). This results in a reduced ability to cope independently (impaired social functioning), and begins before adulthood, with a lasting effect on development. Disability depends not only on a child’s health conditions or impairments but also and crucially on the extent to which environmental factors support the child’s full participation and inclusion in society” [1] (para. 1,2).

ID is more common in low- and middle-income countries (LMIC) than in wealthier countries [2,3,4,5,6,7], for a range of reasons, including increased risk factors such as poor nutrition, poverty, violence, increased exposure to environmental toxins and substance abuse during pregnancy, amongst others, and there are fewer specialised intellectual disability services in these countries to support people with ID and their families [3,8,9]. Specialised intellectual disability services refers to the services designed specifically for those diagnosed with ID. High prevalence rates, scarcity of resources and limited research studies for People with Intellectual Disability (PWID) in South Africa, make a strong motivation for the current study. An article setting global priorities for research into developmental disabilities [10] suggests, among other recommendations, that more needs to be done to make health systems more accessible to families of children with ID. Some research suggests, however, that even in contexts where health services are in reasonably close proximity for families, there may be under-utilisation of these services by those who could potentially benefit from them [9,11,12]. However, most of these findings were based on hospital records and folder notes where, in some cases, there was no diagnosis available. A gap still exists regarding the subjective experiences, views and perceptions of caregivers and parents themselves of children with ID in terms of their failure to use the services for which they are eligible.

There is evidence in the literature that, among other things, stigma and discrimination have contributed to barriers to healthcare in many parts of the world [13,14,15,16,17,18]. PWID still face social exclusion and discrimination more than those without ID [14,19,20,21,22]. As a result, more than the general population, they may face substantial difficulties in areas of health, education, housing and employment [23,24,25,26]. ID is widely known as a stigmatising condition, however, research in this area, especially in the Global South, is still very sparse [3,27]. Although South Africa is still the only country in Africa with inscribed constitutional rights for people with disabilities, implementation remains a major challenge because of lack of resources [3]. While studies have shown that public attitudes, stigma and discrimination can act as potential barriers to healthcare access for PWID and their families, it is not clear whether this contributes to people with ID not using services even when such services are available to them.

There is strong evidence from high-income countries with sound programmes and policies on ID suggesting that many PWID do not use specialised ID services although they are eligible. Records from hospitals and special services data show that there is a significant decline in numbers of PWID attending specialised intellectual disability services in the United Kingdom (UK) [28]. Reasons for this decline amongst minority groups included, but were not limited to, attitudes, beliefs and health professionals’ attitudes towards those from different cultural backgrounds [28], while others reported adjustments related to deinstitutionalisation of individuals with ID to the communities attributable to lack of specialised trained staff in general psychiatric services as well as lack of information about services available for people with ID [29]. The failure to access specialised healthcare services can lead to poor health outcomes. Emerson (2011, p. 155) [30] notes that “as such, the poorer health status of people with intellectual disability can be appropriately described as an example of health inequity”. He further reported that non-users had higher health risks (exposure to material hardships, neighbourhood deprivation and smoking) compared to PWID who used ID services, although the difference was marginal (overall both PWID who accessed services and the ‘hidden’ PWIDs who were not known to ID services had high exposure to health risks/social determinants of health). 

In addition, Boag-Munroe and Evangelou [31] conducted a systematic review on challenges related to service provision for hard-to-reach families in the Global North (UK, Canada, United States of America, Australia). The authors used various synonyms to define ‘hard to reach’ families, including hidden populations, vulnerable, underserved, fragile families, socially excluded, disengaged, marginalised, non- (or reluctant) user, high risk, at risk, families with multiple or complex needs, minority groups, minority ethnic, ethnic communities and less likely to access services [31] (p. 213). They quote Landy and Menna (cited in Boag-Munroe and Evangelou [31]) who state that “working effectively with families who might be labelled “hard-to-reach” involves a shift from perceiving the family as being “hard-to-reach” to thinking about what makes the service that is being offered hard to accept for a particular family” [31] (p. 180). In the literature reviewed, the authors identify three main organisational barriers to access for hard-to-reach families and hard-to-engage families. The barriers go beyond tangible physical access issues and include alienating attitudes and unwelcoming buildings. The authors acknowledge the latter two as being the most difficult to resolve, requiring money and time to overcome. Barrier 1 relates to communication, barrier 2 is about setting and barrier 3 has to do with quality of service. They highlighted one of the key challenges with the setting as lack of awareness by services that families may have valid reasons for non-engagement and presupposing families’ needs. Physical location is also mentioned as a setting barrier where hard-to-reach families face obstacles in accessing a service because it is spatially distant from where they are, and the authors suggest it might be useful to ‘shift locus of provision towards the community’ to increase visibility and improve access [31] (p. 218). The literature review findings also indicate that families might perceive settings and services as stigmatised or stigmatising.

As part of a larger study on caregiving of children with ID in urban Cape Town, South Africa [32], we became interested in a sub-group of families who had never used the ID services available to them, or who had stopped using them. The objective of the study is to explore their subjective experiences of the ID services and their reasons for not using them. We report here on interviews with this small group of families.

## 2. Materials and Methods 

### 2.1. Research Design

To explore the views and perspectives of parents and caregivers of children with ID who are not using specialised hospital services, a qualitative research design was employed [33]. Based on Kleinman’s Explanatory Models (EMs) framework [34], we developed an interview guide to help explore caregivers’ and parents’ views. This was an appropriate form of design to explore subjective experiences of caregivers and parents of children with ID who are not using hospital specialised intellectual disability services. A Consolidated Criteria for Reporting Qualitative Studies (COREQ), a 32 item checklist (Appendix A) was also used to ensure proper and valid reporting on all aspects of the study [35]. 

### 2.2. Study Setting

The study was conducted in Khayelitsha, a predominantly Black (The use of ‘racial’ terminology in South Africa, as elsewhere, is highly politicised and contested [36]. We do not claim that any of the terms we use, such as ‘White’, ‘Black’, ‘Coloured’ or ‘Indian’, which are still terms used in contemporary South African equity legislation, and have strong social (if contested) meanings, have any basis as biological or scientific categories. They are social categories which have proven remarkably durable, even in a supposedly non-racial democracy [36,37]. The term ‘Black’ in South African terminology refers chiefly to people who speak indigenous languages; ‘Coloured’ refers to people of diverse origins, most having Afrikaans as a first language. Under apartheid, the Western Cape Province was deemed a preferential area for ‘Coloured’ labour and settlement, and ‘Black’ people, even those born in the province, were regarded as foreigners. Despite the end of apartheid, and despite the widespread recognition that ‘racial’ labels do not refer to ‘real’ differences in any scientific sense, the fact remains that social stratification along ‘racial’ lines is an ongoing reality [36]. In particular, despite the fact, for example, that isiXhosa is an official language in South Africa and in the province where our study was conducted, patients still struggle to access culturally appropriate care in their home language [38,39,40]) township in Cape Town, South Africa. The township was chosen because it represents a fairly typical urban settlement in South Africa, a Black-dominant township in South Africa that remains disadvantaged and largely segregated from other parts of society [41]. The specialised ID services for this area are allocated in a nearby psychiatric hospital. The hospital was built during the apartheid period to serve the Coloured community exclusively. However, the hospital is now open to all service-users and most people from the neighbouring communities, including Khayelitsha, a large area of mainly informal dwellings, live in the catchment area of the hospital provides [42].

### 2.3. Recruitment and Sampling

As part of our larger study, we recruited eight Black isiXhosa speaking parents and primary caregivers of children with ID who were not using specialised ID services. Parents and caregivers who were currently using the service were excluded. Participants were sampled and recruited using purposive sampling methods. Through the help of those using the hospital services, and through the assistance of cultural and spiritual leaders in the system, participants were identified through contacts and snowballing. As the non-users of services are, by definition, a hard to reach population, we used all the contacts we could to identify potential participants. These potential participants were then contacted via telephone by the first author, who speaks isiXhosa as his first language, and this made it easier to build rapport with the participants, while remaining mindful of the possibility of over identifying with participants. We then contacted the parents or caregivers and requested to visit them at their homes to speak with them. As indicated above, participants lived in Khayelitsha and surrounding areas. Khayelitsha is one of the largest townships in South Africa, mainly impoverished with a high proportion of informal (shanty) housing in the Cape Flats in Cape Town.

### 2.4. Data Collection

We conducted the individual interviews between April and May 2018 at the participants’ homes. Participants were not participating in any services the healthcare facility provides and they preferred to have interviews at their homes in Khayelitsha. Kleinman’s [43] EM framework to develop a semi-structured interview guide was used and constructed in English. Following, this the guide was then translated into isiXhosa by the language and communication centre at Stellenbosch University. This was necessary in order to minimise any language barrier because all participants spoke and understood isiXhosa as their first language. After the original translation, the first author, who speaks and understands isiXhosa as his first language, did minor edits. Following this, all interviews were conducted by the first author who speaks isiXhosa as his first language and qualified as a clinical psychologist with over ten years of experience of working in ID services. The guide was then tested before it was administered to collect data. We recorded all interviews using an audio recording device and all the necessary permissions of the participants were sought. The data presented here concern questions about accessing (or not accessing) the nearby ID services.

### 2.5. Data Analysis

Once interviews were completed, we transcribed the audio recorded interviews in isiXhosa and the transcriptions were then translated into English by a language translator who also speaks and understands isiXhosa as his first language. Following this, the first author, who speaks isiXhosa as his first language, then checked the transcripts against the original recording to ensure accuracy. Thereafter, the first author performed an initial analysis of data which was then checked in collaboration with the second author for accuracy. When disagreements emerged, we vigorously discussed these until a resolution was reached. Following Braun and Clark’s [44] guide, we used thematic content analysis to analyse the data and codes were categorised into themes.

### 2.6. Ethics

Ethical approval was sought and obtained from the Stellenbosch University Humanities Research Ethics Committee and the Western Cape Department of Health Ethics Committee before the data collection process. Formal permission was also sought from Lentegeur Hospital Research Committee.

Following ethical guidelines, participants who showed signs of distress following the interviews were referred for individual psychological support or to a parent support group at Intellectual Disability Services (IDS), Lentegeur Hospital (LGH). Children of the interviewed participants were already known to the local department of social services and various social agencies operating in the communities providing social support and guarding against any form of abuse. They were also mainly receiving care dependency, disability or childcare grants from the Department of Social Services. 

## 3. Results

Participants gave a number of reasons as to why they are not using the ID services close to them. These included financial difficulties, fragile care networks and opportunity costs, community stigma and lack of safety, lack of faith in services and powerlessness at effecting changes and self-stigmatisation. We present data on each of these in turn (we used pseudonyms to protect the identity of the participants).

### 3.1. Financial Difficulties

Though the high level of care available at the specialist ID services is offered free of charge to those who cannot afford to pay, there are other financial barriers which affect caregivers’ ability to access services. One participant decided not to use the services anymore because she had no money to pay for the public transport. The whole family depended on the state-provided Child Support Grant of R420 (approximately $25) per month because the mother, who is a single parent, was not working and had no other source of income:

* I stopped taking him there in 20 what? [20]16. I think in 2016 because I was not working that time, so I had to borrow money in order to go there and then I couldn’t pay it back. That time my allowance [the value of the Child Support Grant at the time] was about R260, and with that amount I had to buy food and … so I was spending a lot on travelling fees to go there.*

(Nosakhele)

Other parents shared similar difficulties:

*He receives the same grant that other children who do not have disability get. I mean there’s nothing … He receives the same grant as other kids. Only that R420.*

(Nolitha)

*He does not receive Disability Grant … Yes, he just receives Child Support Grant of R400, he doesn’t receive the Disability Grant.*

(Nosipho)

The caregivers here are referring to a grant which is available from the state for care of children with disabilities, and this is the Care Dependency Grant of R1860 (approximately $110) per child per month, commonly referred to as a ‘disability grant’. Despite being eligible to receive this grant, which is substantially bigger than the child support grant, parents were not accessing it for various reasons:

*At his school they said they are going to call us … Last year we were called to come for a meeting at the school because people from SASSA [South African Social Security Agency, the agency which administers grants] will come and register children who does not receive disability grant. Till now they haven’t [inaudible].*

(Nosipho)

*I’m waiting for transport that will fetch me and the child who was burnt [and disabled]. It never came. I waited for it until forever. Those ladies from social services said last time they called they said another child who was at hospital passed away so they went to that child’s funeral. After the funeral they will call me back and inform me when they will come.*

(Nolitha)

It appears that a combination of logistical challenges from government agencies, and transport costs to go to the relevant office to register to receive a Care Dependency Grants, are major barriers for our participants. In the absence of the Care Dependency Grant, the cost of getting to the hospital (and ID services) is unaffordable.

### 3.2. Fragile Care Networks and Opportunity Costs

For some participants, a change in those acting as primary caregivers for the child affected their use of services. For example, one family stopped using the services because the primary carer of the child passed away. After the death of the primary carer, those who were left behind to care for the child did not take the child for his follow-up appointments because they were working and could not get time off work. They had no one else to assist with taking the child to ID services:

*My mother used to take him to the hospitals that had children with the same condition as his. When she passed away, I had no one else to help to take him for his appointments.*

(Lulama)

### 3.3. Community Stigma and Lack of Safety

Some caregivers stopped taking the children to ID services because of community stigma in the context of high levels of violence in the community. Some caregivers feared for the safety of the child, or worried that the child might be taken advantage of if she left the home. As one carer put it:

*Eh, I think the reason why I didn’t want her to go to school or hospital was because I was fearing that other people won’t understand that she is a girl child with a disability. People outside are very cruel. And secondly, they will see someone and think that they are seeing a lady but they are seeing someone with [a] disability [this is a reference to the sexual maturity of the child, and a concern about gender-based violence]. And thirdly why I wanted to keep her indoors was because she seemed much more safe indoors than to be exposed outside.*

(Lungelwa)

Another carer who preferred to keep the child away from others said:

*Some people, sir, tease this girl saying she is a creature [an animal]. Because she walks differently. I go outside to fight with whoever is teasing her. I tell them that it’s not this child’s fault that she is like this.*

(Linda)

Stigma affected relationships within the family, and even where caregivers and children could live:

*I just moved in to my stepmother’s house but my stepmother started gossiping about my child’s disability to her neighbours and colleagues. I was then forced to move out of the house and came here. I couldn’t continue staying with them having a child with this condition.*

(Nokhanyo)

There were also concerns that as a result of the child’s disruptive behaviour, others were not tolerant, which exacerbated stigmatisation and led caregivers to be more careful about keeping the child out of public view:

*He doesn’t play with other children, he fights with them. So that is why I’m saying, it’s because of the teasing that is happening around him. Because of this he doesn’t play much with children of his same age, he plays with children that are younger than him. He hits children that are the same age as him, but plays well with children of two to three years that are younger than him.*

*Every day I receive reports about him from the community and sometimes I don’t know how to handle those situations although they know what kind of a child he is, you understand. So when their parents come to my house to confront me, it becomes difficult for me to explain his condition to them.*

(Akhane)

### 3.4. Lack of Faith in Services and Powerlessness at Effecting Changes

Not taking the child to ID services was part of a pattern, for some, of not taking the child to school or other services, consequent, it appears, on the belief that these services could not help and would just add to stigma:

*He does not go to school because there’s nothing he’s going to learn because he is just sitting … I’ve never … maybe I’m not ready because you know people, they like to judge others. So I don’t want them to go there and I don’t … They know I have a son because I talk about my son but they never came here to see him. It’s few of them that do come but I don’t go around encouraging people to come and see him. So, when you hear things that come out of people’s mouth you would weigh what they say. And then for you to not get hurt you stay away from them.*

(Bulelwa)

One mother believed that her child’s ID was caused through preventable birth trauma, and she blamed the hospital where she delivered the child for this. She had, however, decided not to claim compensation from the hospital, on the advice of another doctor who told her the attempt could be enervating and fruitless.

*So that doctor said suing the hospital is going to take your time and energy and you won’t have time for your child. It’s better to love your child and accept his condition. Because the moment you go up and down you are going to chase after money and forget about your child.*

(Nosiphiwo)

This generalised, for her, to a lack of faith in whether services could be trusted to help her and her child.

### 3.5. Self-Stigmatisation: Feelings of Incompetence and Guilt

Some caregivers felt unequal to the task of caring for the child, and feared being exposed if they went out with the child. One mother, for example, mentioned her young age and those of other parents:

*A child with disability is an everyday challenge. That’s why I don’t blame mothers who neglect their own children. So that’s why other mothers decide to give up on them. More especially when you’re young. I’m also young; don’t be misled by this doek I’m wearing. But having an experience of a child … Because I had my child when I was only 17.*

(Likhanye)

Guilt was also a factor keeping parents away from services:

*I felt bad because he was my first child. So bad because first time I have a child and have a child with this problem. I was feeling very guilty.*

(Uzusakhe)

*I’m also to be blamed for what went wrong, I always punish myself with that. I think I’m punishing myself for my mistakes. So I always blame myself that I’m the cause of his disability, you understand. I feel guilty and blame myself because I did not accept him at first.*

(Nosolule)

## 4. Discussion

The current study explored lived experiences of parents and caregivers of children with ID who are not attending specialised intellectual disability services for their children’s needs. Despite living reasonably close to specialist ID services, these caregivers were not using the services. Common themes identified included financial difficulties, fragile care networks and opportunity costs, community stigma, lack of safety, lack of faith in services and powerlessness at effecting changes, self-stigmatisation, feelings of incompetence and guilt. 

A striking feature of these findings, consistent in almost all of what parents said, is the multidimensionality of urban poverty in a middle-income country. Most fundamentally, caregivers could not afford the transport costs to take their children to services. These findings are similar to other studies that investigated barriers to care access for people with ID in LMIC. Most of these studies found that services for people with ID were often located at great distances from the service users and most of them could not afford transport costs to access healthcare facilities [13,45,46]. An aspect of their inability to afford transport is the fact that, in the absence of any general social security unemployment grant in South Africa, and in the context of endemic unemployment, whole families in South Africa subsist on meagre old age pensions, disability pensions and child grants [47,48]. We can see in the data a vicious cycle of poverty—despite these caregivers being potentially eligible to receive a Care Dependency Grant, due to the cost and logistics of registering to receive the grant, they could not attain the grant. This fact in turn seems to have been a barrier to using the ID services, as the Child Care Grant, often the only income in the household, was so meagre [48].

Poverty, however, is more than lack of income. It also has implications for social exclusion, exposure to climate and other negatively impactful factors, as well as to violence [49,50]. There are higher rates of mortality in poorer areas; which, as the data show, can have implications for caregiving. An aspect of the caregivers’ lack of use of services seems to relate to a sense of a lack of agency as well as a lack of faith that institutions, such as schools or hospitals, have the potential to make a difference in the lives of the caregivers and the children with ID. In literature on access to biomedical services for families with a child with ID in Africa, cultural reasons are often cited as why people do not use services [51]. In our study, no caregiver cited religious or cultural barriers to using care. The focus was predominantly on social isolation, the cost and difficulty of accessing care and the sense that services might not be able to help. Though this lack of faith in services may in part be explained by participants’ explanatory models of ID as not changeable [32], there seems also to be a more general feeling of isolation and of being cut off from services.

This isolation is exacerbated by the pervasiveness of stigma within households and beyond. Though there is evidence for the stigmatisation of ID globally [52], the lived experience of stigmatisation may be more impactful in a context in which people live in very small dwellings, often with shared taps and toilets. There is no way to conceal taking a child to ID services in an area in which there is very high population density and much of life is lived on the street in view of neighbours. This is very different to a middle-class existence.

Studies from other parts of the world have shown that although many PWID and their families were eligible for specialised ID services, they did not all make use of these services [28,30,31,53,54]. It is difficult to establish the magnitude of under-utilisation of services, and in the South African context where there are no systems in place to track attendance, as well as non-attendance of the service users, it is not possible to generate comparative data from information readily available. Financial difficulties have been reported as potential barriers to healthcare access for marginalised population groups in a number of African countries [13,17,55,56]. However, none of these studies reported service users giving up permanently on using the services, as some of our participants seemed to have done. In South Africa, much work has been done on making services more affordable, but what is clearly needed is an integrated support system that goes beyond offering services at the point of care. Most South Africans, most of whom are Black, face structural disadvantages where their lives are challenged by systematic barriers [57]. These barriers to basic healthcare needs for parents and caregivers and their children with ID are indicative of a much broader systematic disadvantage that exists in South Africa. Specialised intellectual disability services should consider the access needs of low-income primary caregivers of children with ID from the minute they step out of the home, not only once they reach the service. This finding also reinforces the point made by Boag-Munroe and Evangelou [31] regarding the need to shift services to communities where families with children with ID reside, in order to improve access. 

## 5. Limitations

There are potential limitations to this study. First, the study included a small sample and therefore the results may not be generalised. Secondly, we restricted the study to one urban context in Cape Town, South Africa, and there may be other issues in other contexts. Thirdly, only parents and caregivers were interviewed and not the children themselves.

## 6. Conclusions

The challenge of creating services which people are able to use goes far beyond setting up services and waiting for clients to arrive. It must be possible to get to the services, and the services themselves need to be welcoming and not alienating [31]. These issues, as we have seen, go far beyond healthcare-related issues as narrowly understood. If children with ID are to receive the best healthcare they need, social changes far beyond the domain of the hospital are needed. The very social conditions which create a greater risk of ID in poorer communities act as barriers to receiving the best care available. It is noteworthy that priorities for research into improving ID services in low-income contexts tend to focus on the improvement of health services and systems [10]; our data suggest that broader social conditions are as important, or possibly even more important, as a focus of concern.

## Appendix A

**Table A1:** Consolidated criteria for reporting qualitative studies (COREQ): 32-item checklist.

No. Item	Guide Questions/Description	Reported in Section
**Domain 1: Research team and reflexivity**		
*Personal Characteristics*		
1. Inter viewer/facilitator	Qualified clinical psychologist	Methods
2. Credentials	MA, Clinical Psychology	Methods
3. Occupation	Clinical Psychologist	Methods
4. Gender	Male	N/A
5. Experience and training	11 Years, Clinical Psychologist	Methods
*Relationship with participants*		
6. Relationship established	No	N/A
7. Participant knowledge of the interviewer	No prior relationship	N/A
8. Interviewer characteristics	Interest in the research topic; isiXhosa-speaking person working in ID services	Methods
**Domain 2: study design**		
*Theoretical framework*		
9. Methodological orientation and Theory	Kleinman’s Explanatory Models	Methods
*Participant selection*		
10. Sampling	Purposive and snowballing	Methods
11. Method of approach	face-to-face, email	Methods
12. Sample size	Eight	Results
13. Non-participation	0	Methods
*Setting*		
14. Setting of data collection	Participant’s home	Methods
15. Presence of non-participants	No	Results
16. Description of sample	All women, isiXhosa speaking and have a child with ID.	Results
*Data collection*		
17. Interview guide	provided	Methods
18. Repeat interviews	No	N/A
19. Audio/visual recording	Yes, audio recording	Methods
20. Field notes	Field notes were uses	Methods
21. Duration	Interviews were roughly 60 minutes	Methods
22. Data saturation	Yes	Methods
23. Transcripts returned	Yes	N/A
**Domain 3: analysis and findings**		
*Data analysis*		
24. Number of data coders	None	Methods
25. Description of the coding tree	No	N/A
26. Derivation of themes	Yes	Methods
27. Software	Nil	
28. Participant checking	No	Strengths and limitations
*Reporting*		
29. Quotations presented	Yes	Results
30. Data and findings consistent	Yes	Relationship to existing knowledge
31. Clarity of major themes	Yes	Results
32. Clarity of minor themes	Yes	Discussion
